# Polymorphisms of the *ICAM-1 *exon 6 (E469K) are associated with differentiation of colorectal cancer

**DOI:** 10.1186/1756-9966-28-139

**Published:** 2009-10-13

**Authors:** Qing-lei Wang, Bing-hui Li, Bin Liu, Ya-bin Liu, Yue-Ping Liu, Sui-Bing Miao, Yi Han, Jin-Kun Wen, Mei Han

**Affiliations:** 1 Department of Biochemistry and Molecular Biology, Institute of Basic Medicine, Hebei Medical University, No 361, Zhongshan East Road, Shijiazhuang, 050017, China; 2 Department of Surgery, Fourth Hospital, Hebei Medical University, Shijiazhuang, 050011, China

## Abstract

**Background:**

Genetic factors are thought to play a role in development for colorectal carcinogenesis. *ICAM-1 *is a polymorphic gene, thus, the present study investigated the relationship between the polymorphisms of *ICAM-1 *and the susceptibility and phenotypical characteristics of colorectal cancer (CRC).

**Methods:**

The polymorphisms at *ICAM-1 *exon 4 (G241R) and exon 6 (E469K) were detected by PCR with sequence-specific primers. The relationship between specific genotypes of *ICAM-1 *and differentiation of CRC was evaluated by the histological grade.

**Results:**

We showed only GG genotype of *ICAM-1 *individuals in either CRC or normal controls. The KK genotype of *ICAM-1 *K469E was found more frequently than in the controls (*P *< 0.05). Patients with well-differentiated CRC displayed the KK more frequently than those of poor differentiation (*P *< 0.05).

**Conclusion:**

The findings indicate that polymorphisms of G241R are rare in Chinese population and that KK genotype of *ICAM-1 *K469E is significantly associated with well differentiation of CRC.

## Background

Colorectal cancer (CRC) is a common malignant disease around the world. CRC has an extremely poor prognosis owing to insidious symptomatology, late clinical presentation and rapid progression and so lead to poor 5-year disease-free survival [1,2]. CRC is related not only to living habits such as dietary but also to the susceptibility of heredity [3]. Individuals who have first-degree relative with CRC have the increased risk of the CRC compared with those without a family history [4], suggesting that genetic factors contribute to risk for colorectal carcinogenesis [5].

The intercellular adhesion molecule-1 (ICAM-1) is a single-chain cell surface glycoprotein that belongs to the immunoglobulin superfamily. It is known that ICAM-1 can be aberrantly expressed in CRC and suppress cancer progression via activation of the host immune surveillance system and prevention of cells from detaching from the primary tumor mass and thus attenuate or eliminate metastasis [6,7]. Two single-base polymorphisms in human ICAM-1 gene have been reported, in exon 4 and 6, changing codons 241(G241R) and 469(K469E), respectively, which are common genetic variations associated with diseases [8]. However, it is not well documented that the association of the ICAM-1 gene polymorphisms with CRC development. In present study, we analyze the association between the polymorphisms at exon 4 (G241R) and exon 6 (E469K) of *ICAM-1 *and CRC susceptibility and in vivo differences in ICAM-1 level and differentiation in tumor tissues of patients with CRC. Our results suggest that tumor cell differentiation may be influenced by genetic variation in *ICAM-1 *in Chinese population.

## Materials and methods

### Study population

87 cases were patients with a new diagnosis of colorectal adenocarcinoma attending a Hebei Medical University Forth Hospital, China between December 2007 and August 2008. 102 volunteers without CRC were used as controls. The average age of the subjects was 55 years (range, 34-83 years). The peripheral blood specimens from patients with CRC and controls were collected at the time of the diagnosis after informed consent was obtained. All the tumor and matched normal tissues investigated in this study were obtained from patients who had undergone a surgical resection. The diagnosis and staging of CRC were assessed according to the WHO classifications [9] and TMN classifications [10]. The study was approved by the institutional research board at Hebei Medical University.

### Genotyping of ICAM-1 gene polymorphisms

Genomic DNA was extracted and purified from whole blood lymphocytes using a blood DNA Kit (Omega Bio-Tek Co., USA) according to the manufacturer's instructions. PCR with sequence-specific primers (SSP) was used to detect the *ICAM-1 *polymorphisms at Exon 4 (G241R) and Exon 6 (E469K) as described elsewhere [11,12]. For G241R in exon 4, two sequence-specific forward primers: 5'-GTGGTCTGTTCCCTGGACG-3'(G241) and 5'-GTGGTCTGTTCCCTGGACA-3' (R241), and for K469E (exon 6) two sequence-specific reverse primers: 5'-GCACATTCACGGTCACCTC-3' (K469) and 5'-GCACATTCACGGTCACCTT-3' (E469) were used. Each combination of the four primers contained one forward primer and one reverse primer for the 927 bp fragment of sequence-specific amplifications, respectively. For the positive internal control, the primers 5'-GAAGGTGAAGGTCGGAGT-3'(forward) and 5'-GAAGATGGTGATGGGATTTC-3' (reverse) coding for the 225 bp fragment of human glyceraldehyde-3-phosphate dehydrogenase (GAPDH) gene were used.

PCR was performed in a final volume of 20 μl in 96-well plates. The final concentrations of the reagents were as follows: 200 μM of each dNTP, 2.5 mM MgCl_2_, 0.5 μM of each primer, polymerase buffer, between 0.01 and 0.1 mg DNA, and 0.2 units of Taq polymerase (Promaga). The PCR cycle conditions were 94°C for 4 min, followed by 35 cycles at 94°C for 30 s, 69°C for 45 s and 72°C for 40 s, with a final extension step at 72°C for 10 min. 927 bp of PCR product was identified by gel electrophoresis on 2% agarose gels stained with ethidium bromide.

### ICAM-1 expression analysis

Western blot analysis was used to detect ICAM-1 protein expression in both tumor and matched adjacent normal tissues from patient with CRC as described previously [13]. The tissue lysates were prepared from the colorectal tissues [14]. Equal amounts of proteins were separated by electrophoresis on an 8% SDS-polyacrylamide gel and then electrophorytically transferred to polyvinylidene difluoride membranes (Millipore Co, Billerica, Massachusetts, USA). The membrane was incubated with anti-ICAM-1 antibody (1:1000; Santa Cruz), followed by a secondary anti-rabbit antibody (1:20000; Santa Cruz) using chemiluminescence protocol (Santa Cruz).

### Immunohistochemistry analysis

Immunostaining of sections from CRC tissues was performed with the anti-ICAM-1 (1:200) as described previously [15]. Staining intensities were determined by measuring the integrated optical density (IOD) with light microscopy using a computer-based Image-Pro Morphometric System by two independent observers in a double-blind manner.

### Statistical analysis

Each polymorphism was tested in controls to ensure the fitting with Hardy-Weinberg equilibrium. To test the hypothesis of association between genetic polymorphisms and CRC, multivariate methods based on logistic regression analyses were used. Allele and genotype frequencies in all subjects were calculated by direct counting. Hardy-Weinberg equilibrium was tested using the Fisher's exact test. The strength of the gene-cancer associations was measured by odds ratio (OR) and its 95% confidence interval (CI). *P *< 0.05 was considered statistically significant. The SPSS was used in the statistical analysis.

## Results

### Polymorphism of ICAM-1 K469 E may be associated with CRC risk

**Figure 1:**
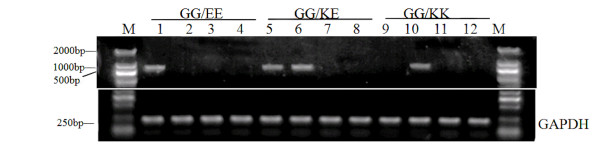
***ICAM-1* G241R and K469E genotypes**. Lane M: Marker; Primers: G241-E469 (lane 1,5,9); G241-K469(lane 2,6,10); R241-E469(lane 3,7,11); R241-K469 (lane 4,8,12).

The polymorphisms of *ICAM-1 *in all cases and controls are shown in Table 1, which were conformed to Hardy-Weinberg equilibrium (*P *> 0.05). In either CRC cases or controls, only GG genotype of *ICAM-1 *exon 4 (G241R) was identified, while the exon 6 (K469E) homozygous and heterozygous individuals were observed (Figure 1). The distribution of the *ICAM-1 *K469E genotypes was significantly different between CRC cases and controls (*P *< 0.05). In 102 controls, the K allele frequency was 63.73%, which is different from that in the cancer cases (73.56%). Subjects with K allele in CRC had a 1.58-fold increase, compared with controls (*P *= 0.041). K allele was significantly associated with a increased risk of CRC (OR = 1.58, χ^2 ^= 4.194, 95% CI, 1.02~2.46, *P *= 0.041). The frequency of KK genotype in CRC cases was more than that in the controls (57.47% vs 42.16%, χ^2 ^= 4.406, *P *= 0.036). Subjects with KK genotype had a 1.85-fold increase in CRC risk compared with those with KE+EE genotypes.

**Table 1:** Allele and genotype frequencies of the ICAM-1 K469E polymorphisms in CRC cases and controls

	**CRC (n = 87) (%)**	**Controls (n = 102) (%)**	***P***	**OR (95% *CI*)**
Genotype				
KK	50 (57.47)	43 (42.16)		
KE	28 (32.18)	44 (43.14)	0.036^a^	1.85 (1.04~3.31)^b^
EE	9 (10.35)	15 (14.7)		
Allele				
K E	128 (73.56) 46 (26.44)	130 (63.73) 74 (36.27)	0.041	1.58 (1.02~2.46)^c^

OR, odds ratio; CI, confidence interval.

a, Genotypes: KK vs KE+EE.

b, OR for KK vs KE+EE genotypes in CRC.

c, OR for K vs E allele in CRC.

### Polymorphism of ICAM-1 K469E is associated with tumor differentiation

**Figure 2:**
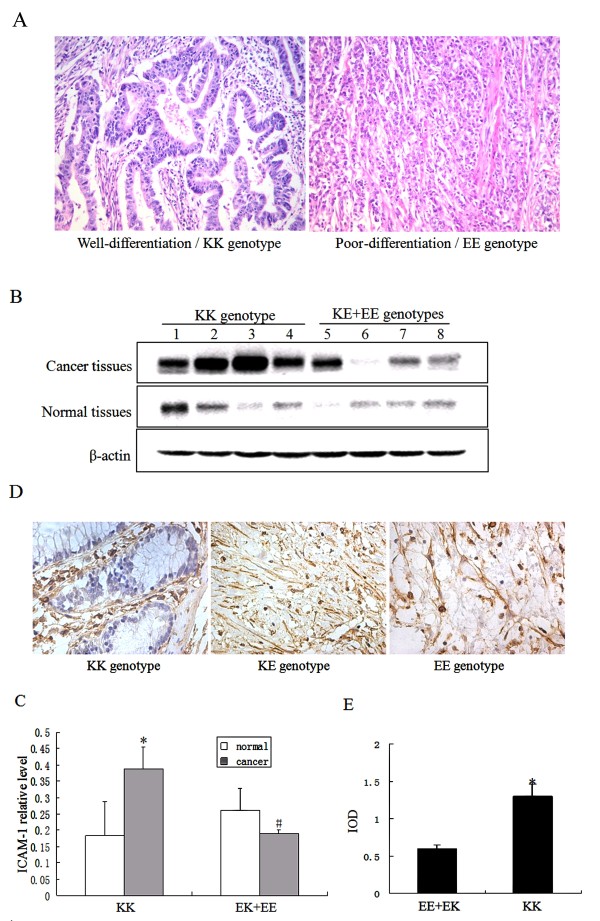
**Polymorphism of *ICAM-1* K469E is associated with cancer differentiation and ICAM-1 expression in CRC**. (A), Representative histological sections of CRC with KK and EE genotypes (Magnification, × 400); (B), Western blot analysis for ICAM-1 expression of CRC with KK, KE and EE genotypes; (C), Densitometric scanning of Western blots, n = 15, * *P *< 0.05 vs controls; # *P *< 0.05 vs CRC with KK genotype; (D), Representative ICAM-1 staining of the cross sections of CRC with KK, KE and EE genotypes (Magnification, × 400); (E), Average IOD of ICAM-1 staining of CRC cross sections (n = 15). IOD represents relative ICAM-1 protein level in tumor tissues. * *P *< 0.05 vs KE+EE genotypes.

The potential associations of the *ICAM-1 *K469E genotype with tumor characteristics are presented in Table 2. No correlation was found between K469E genotypes and tumor location, presence of lymph node metastases, Dukes stage, or age and gender at diagnosis. The KK genotype was more frequently found in cases with a well-differentiated CRC (*P *= 0.033) (Figure 2A and Table 2), although with the increased CRC risk. In contrast, the tumor tissues from the cases with KE+EE genotype showed poor differentiation compared with those with KK genotype (*P *< 0.05). The results suggest that there is correlation between the K469E genotype and the phenotypical characteristics of CRC.

**Table 2:** Distribution of various genotypes of ICAM-1 K469E in relation to clinicopathological and other variables in CRC cases

**Variables**	**Cases (n)**	**KK**	**KE+EE**	***χ*^2^**	***P***
Age					
≤ 55	27	16	11	0.051	0.821
> 55	60	34	26		
Gender					
Male	49	28	21	0.005	0.944
Female	38	22	16		
Tumor location					
Colon	30	14	16	0.004	0.95
Rectum	57	27	30		
Differentiation					
Well and moderately	62	33	29	4.564	0.033
Poorly	25	7	18		
Metastasis					
No	75	41	34	1.75	0.186
Yes	12	9	3		
Dukes stages					
A+B	50	30	20	0.308	0.579
C+D	37	20	17		

### KK genotype is correlated with increase in ICAM-1 expression in tumor tissues

We next set out to assess whether the K469E genotype is correlated with differences in ICAM-1 expression using lysate extracted from the tumor and matched adjacent normal tissues of CRC patients with KK or KE+EE genotypes. There were no differences in ICAM-1 level in matched normal tissues of all tested patients. KK genotype patients showed an increase in the expression of ICAM-1 protein in tumor tissues relative to the matched normal tissues (*P *< 0.05, Figure 2B and 2C). However, the difference of ICAM-1 level between tumor and matched normal tissues was not observed in the patients with KE+EE genotypes. Meanwhile, ICAM-1 level was higher in the tumor tissues of individuals with KK genotype than that of the KE+EE genotypes (*P *< 0.05). We also observed that the distribution of ICAM-1 was exclusively extracellular in all colorectal tumors (Figure 2D and 2E). Taken together, these results indicate that ICAM-1 protein is accumulated in CRC tissues with KK genotype.

## Discussion

Polymorphisms of *ICAM-1 *K469E and G241R are common genetic variation in populations and associated with several autoimmune diseases, such as multiple sclerosis, type 1 diabetes, or Crohn's disease [12,16,17]. In current study, we have found only GG genotype individuals in either CRC cases or normal controls. The variants in G241R were not observed in our tested population, suggesting that the polymorphisms of G241R may be rare in Chinese, consistent with the Japanese and Koreans, respectively, probably reflecting that there is a common ancestor in these populations [16]. Our observation is different from the previous study concerning the G allele frequency in European-American population that showed less G allele frequency (0.796-0.971) [12,18,19]. The distribution of K469E genotypes and allele frequencies in exon 6 of the *ICAM-1 *was significantly different between CRC patients and controls, and between patients with well differentiation and poor differentiation of tumor tissues. In CRC patients, the KK genotype was found more frequently than in the controls. The previous studies have shown that the K allele frequency is 0.437-0.630 in different populations [16,20]. The KK genotype frequency in patients with well-differentiated tumor tissues was more than that in those of poor differentiation. Although the significance and the functional or therapeutic relevance of our findings remain to be elucidated, the most important finding is that the poor prognosis of CRC seems to be associated with allele E.

Although it is unclear how the *ICAM-1 *K469E polymorphism contributes to the pathogenesis of CRC, we found that the increase in ICAM-1 expression was accompanied by well-differentiation in tumor tissues of KK genotype patients. ICAM-1, as a surface glycoprotein, is expressed on vascular endothelium, macrophages, and activated lymphocytes, and mediates leukocyte circulation and extravasation from the blood into the areas of inflammation and macrophage differentiation [21-23]. The epithelial cells of adult colon do not normally express ICAM-1 which can be expressed subsequent to malignant transformation [24,25]. ICAM-1 expression decreases CRC metastasis and suppress cancer progression via promoting tumor cell motility and attachment to the extracellular matrix [6]. The previous study has showed that expression level of ICAM-1 is high in well differentiated tumor cells and low levels in poorly differentiated cells, and demonstrated a mechanism whereby ICAM-1 expression promotes CRC differentiation and retard metastasis [7]. ICAM-1 plays a role in promoting lymphocyte-mediated tumor killing [26], and this occurs as a result of enhanced binding of peripheral blood mononuclear cells to the tumor cells and subsequent tumor cell lysis [27]. Yet the study suggests that ICAM-1 enhances tumor cell attachment to the extracellular matrix by promoting motility in the context of remodeling, and appears to be acting as a morphogen [7]. These findings provide a possible reason why increasing of ICAM-1 expression occurs in well differentiated CRC tissues.

## Conclusion

Our study herein provides a potential genetic factor for the differentiation of CRC that correlates with *ICAM-1 *K469E polymorphisms because of different ICAM-1 expression. However, we are unable to define the association of the *ICAM-1 *K469E polymorphisms with CRC risk owing to the limitations of the size of the CRC and control populations in the present study. Our findings may help to evaluate the prognosis of CRC according to the individual genetic background.

## Competing interests

The authors declare that they have no competing interests.

## Authors' contributions

BHL provided funding and the CRC samples and designed research program for this study. QLW, YBL and SBM carried out many of the experiments, and drafted manuscript. YPL carried out immunohistochemistry analysis. YH and BL participated in the design of the study and data interpretation. JKW and MH revised the manuscript. All authors read and approved the final manuscript.
